# Lesbian and bisexual breast cancer survivors’ post-treatment resource needs

**DOI:** 10.1007/s11764-024-01650-y

**Published:** 2024-08-05

**Authors:** Bethany Rhoten, Jennifer M. Jabson Tree, Kurt David, Uli Boehmer, NFN Scout

**Affiliations:** 1 Vanderbilt University School of Nursing, 461 21st Avenue South, Nashville, TN 37240 USA; 2 Department of Public Health, Purdue University, West Lafayette, IN USA; 3 University of California at Davis Betty Irene Moore School of Nursing, Sacramento, CA USA; 4 School of Public Health, Boston University, Boston, MA USA; 5 National LGBT Cancer Network, Providence, RI USA

**Keywords:** Breast cancer survivor, Cancer survivors, Sexual and gender minorities, Patient care planning

## Abstract

**Purpose:**

The purpose of our study was to identify and describe determinants of lesbian and bisexual breast cancer survivors’ post-treatment resources.

**Methods:**

We used a cross-sectional descriptive study design. The data reported here were gathered as part of OUT: The National Cancer Survey, administered electronically from September 2020 to March 2021 via social media and community partners. We used descriptive statistics, Fisher’s exact tests, and thematic analysis to analyze survivor perceptions of information availability, treatment environment, care plans, social support, and health.

**Results:**

Of those who participated in the survey, (N =430) 366 identified as lesbian, and 64 identified as bisexual. Mean age was 58.6 years (range 21 – 91 years). Fewer than 11% of our sample indicated they could find helpful information about being a queer person with cancer during their treatment. Over 75% of our sample that received a cancer survivorship care plan indicated that their plan did not include resources for queer individuals.

**Conclusions:**

Affirming cancer treatment environments and resources tailored to the needs of lesbian and bisexual breast cancer survivors are critical for reducing disparities.

**Implications for Cancer Survivors:**

Survivorship care plans should include resources for queer individuals as a part of holistic cancer care.

**Supplementary Information:**

The online version contains supplementary material available at 10.1007/s11764-024-01650-y.

## Purpose

Recent estimates indicate that there are approximately 150,000 new cancer cases and 48,000 cancer-related deaths among sexual and gender minority (SGM) people [1, 2]. Therefore, cancer is an important issue for lesbian and bisexual people. Despite an increase in awareness of the experiences of SGM cancer survivors, disparities in survivorship experiences and resources persist. The overwhelming majority of survivorship resources designed for those affected by breast cancer are hetero- and cisgender-normative.

Cancer survivorship holistically encompasses the physical, mental, social, and financial effects of cancer, beginning at diagnosis and continuing through treatment and post-treatment life [3]. Yet accessing survivorship care is challenging for lesbian and bisexual (LB) patients. It requires patients and their caregivers to navigate multiple layers of healthcare providers, facilities, and plans of care [4–8]. Survivorship care plans are considered a standard of high-quality oncology care; however, they are not received by all cancer survivors, nor is their quality consistent across care settings.

LB breast cancer survivors may have different resource needs after completion of treatment than heterosexual breast cancer survivors. LB breast cancer survivors have more perceived stress after cancer [9], experience poorer quality of life [8], and have concerns about coordinating post-treatment care [10], cultural competence in survivorship care plans [11], late effects of cancer treatment [12], and family and relationship issues [12].

Care environments that are welcoming and affirming are one critical element in addressing disparities in post-treatment resource experiences. These environments encompass diagnostic, treatment, and follow-up care services, with each new environment providing opportunities for patients to interface with new medical professionals and staff. Oncology provider and staff trainings designed to promote SGM patient-affirming environments and culturally sensitive care are a good initial step in providing a base level of care. Alone they are insufficient for reducing disparities and promoting health-seeking behaviors among SGM patients. However, providing equitable affirming care means that patients may feel heard and understood by healthcare providers and facility staff throughout the trajectory of their care [13–16].

Key determinants of LB breast cancer survivor’s experiences with post-treatment resources after treatment have not been well documented. However, understanding these key determinants and how they relate to LB breast cancer survivor experiences and post-treatment resources could help us better meet the needs of these vulnerable cancer survivors and improve the quality of survivorship.

Intentional deliberate patient-centered care initiated at multiple levels of a care setting and care that includes patients’ sexual orientation and gender identity were identified by one of only a few such studies concerning key determinants of cancer survivorship care coordination [17]. It is possible that other key determinants exist and may include patient perceptions and feelings about cancer treatment, cancer care setting, and types of resources provided. For example, it is possible that if LB cancer survivors perceived their cancer care environment to be affirming and welcoming to those with LB identities, they may also be more tuned in and looking for post-treatment resources. This is yet empirically undetermined.

The purpose of our study was to identify and describe determinants of LB breast cancer survivors’ post-treatment resources and priorities for future resource development. These included perceptions and feelings about their care, care setting, care experiences, and awareness of resources provided.

## Methods

### Data source and sample

The data reported here were gathered as part of OUT: The National Cancer Survey. IRB approval was obtained from the WIRB-Copernicus Group prior to conducting any study activities. During early 2020, we developed and refined a web-based survey for lesbian, gay, bisexual transgender, queer, and intersect + (LGBTQI +) cancer survivors in conjunction with an expert panel composed of clinicians, researchers, and community members. The survey was administered from September 2020 to March 2021 via social media outlets and in collaboration with over 100 community partners. Eligibility criteria included a previous diagnosis of cancer, age 18 years or older, self-identify as LGBTQI + , and currently live in the USA. A summary of findings from the parent study is publicly available [18]. Only responses provided by study participants who self-identified identifying as lesbian or bisexual breast cancer survivors were included in this study. We did not place limitations on reported gender.

### Measures

Demographic characteristic questions included age, gender identity, sexual orientation, race, ethnicity, community type, state of residence, and level of education. Survey questions expanded upon those asked by Margolies and Scout [19] and were developed and refined by the research team in conjunction with community partners. In the findings reported in this manuscript, only responses to closed and open-ended questions concerning cancer survivorship were analyzed (Supplemental Table). The content areas of the closed-ended questions included perception of information availability (two questions), treatment environment (two questions), care plans (three questions), cancer survivor social support (three questions), and one’s health (two questions). One open-ended question was included in order to learn more about participants’ perspectives on post-treatment care plans.

### Analyses

#### Closed-ended questions

Descriptive statistics were used to characterize participant characteristics and responses to closed-ended questions. We used Fisher’s exact tests to compare subgroups with differing experiences (e.g., positive vs negative) and answers to categorical (e.g., yes/no) variables. We analyzed responses from the sample in aggregate as well as according to lesbian or bisexual sexual orientation in order to avoid inappropriate generalization of findings among sexual minority breast cancer survivors.

#### Open-ended question

Responses to the open-ended questions were qualitatively coded using thematic analysis for the purpose of informing the meaning of the quantitative data. Thematic analysis is flexible and emphasizes the systematic organization and interpretation of data by identifying recurring ideas, concepts, or patterns which are then grouped into overarching themes [20]. Two analysts (BR, KD) first read the text independently for familiarity and to achieve immersion, each inductively making an atheoretical list of topics or response types based on their interpretation of participants’ responses. An inductive approach was chosen to avoid fitting the data to researchers’ analytic preconceptions [20, 21]. Independently, the analysts categorized each textual response according to topic or response type. BR and KD then met to compare their code lists and code application in order to generate a consensus code book based on their initial analyses. All participant responses were then independently re-coded by each analyst using the agreed-upon code book. Finally, the analysts compared their coding applications and resolved any coding discrepancies. Codes were organized into thematic categories with appropriate subthemes identified. Themes, subthemes, and supporting quotes were presented to paper co-authors in order to elicit feedback from those familiar with the data. This feedback was incorporated into the final presentation of qualitative data herein.

## Results

### Sample characteristics

Table 1 summarizes the sample. Of the breast cancer survivors who participated in the survey (*N* = 430), 366 identified as lesbian, and 64 identified as bisexual. Mean participant age was 58.6 years with a range of 21–91 years. Survey participants overwhelmingly identified as female (93%), White/European American (82%), and non-Hispanic (95%). The Midwest, Northeast, South, and West regions of the USA were similarly represented in this sample, with slightly more than half of all participants residing in a suburban community. Finally, individuals with at least a college degree or vocational certificate represent 83.4% of the study sample.

**Table 1:** Sample characteristics (*N*** = **430)

	Lesbian, *n* = 366 (85.1%)	Bisexual, *n* = 64 (14.9%)
Age (*n* = 358)		
Mean (SD)	59.5 years (9.3)	53.8 years (12.3)
Median (IQR)	60 years (52.3–66.0)	53 years (47.0–63.0)
Range	21 years–91 years	25 years–78 years
Race*		
White/European American	306 (83.6%)	48 (75.0%)
Another racial identity	34 (9.3%)	5 (7.8%)
Missing data	38 (10.4%)	6 (9.4%)
Ethnicity		
Hispanic	17 (4.6)	2 (3.1%)
Non-Hispanic	311 (85.0%)	56 (87.5%)
Missing data	38 (10.4%)	6 (9.4%)
Gender identity		
Male	0 (0.0%)	1 (1.6%)
Female	346 (94.5%)	55 (85.9%)
Transgender	3 (0.8%)	0 (0.0%)
Genderqueer/gender non-conforming	9 (2.5%)	2 (3.1%)
Nonbinary	3 (0.8%)	5 (7.8%)
Another gender identity	4 (1.1%	1 (1.6%)
Missing data	1 (0.3%)	0 (0.0%)
Region of USA		
Midwest	63 (17.2%)	9 (14.1%)
Northeast	59 (16.1%)	9 (14.1%)
South	67 (18.3%)	16 (25.0%)
West	67 (18.3%)	16 (25.0%)
Missing data	110 (30.1%)	14 (21.9%)
Community		
Rural or remote	44 (12.0%)	13 (20.3%)
Suburban	164 (44.8%)	24 (37.5%)
Urban	94 (25.7%)	17 (26.6%)
Missing data	64 (17.5%)	10 (15.6%)
Highest level of education		
Some high school	2 (0.5%)	3 (4.7%)
High school diploma	7 (1.9%)	3 (7.8%)
Some college or vocational training	42 (11.5%)	0 (0.0%)
College degree or vocational certificate	102 (27.9%)	22 (34.4%)
Graduate school	150 (41.0%)	24 (37.5%)
Missing data	63 (17.2%)	10 (15.6%)

^*^Could select multiple responses 1

### Descriptive quantitative survey data

Table 2 summarizes the quantitative closed-ended questions. More than 85% of survivors (lesbian, 89.1%; bisexual, 85.9%) described their cancer care environment as very welcoming or somewhat welcoming, and greater than 88% (lesbian, 90.7%; bisexual, 82.8%) indicated that they were very satisfied or somewhat satisfied with their overall cancer treatment experience. More than half of participants described their current health as good or better (lesbian, 63.4%; bisexual, 56.3%) with more than 70% agreeing that they are currently able to access the resources they need to maintain or improve their health (lesbian, 77%; bisexual, 71.9%).

**Table 2:** Survey questions and answers

	Lesbian	Bisexual
During my cancer treatment, I could find helpful information about my cancer		
* Strongly agree or Agree*	297 (81.1%)	50 (78.1%)
* Neither agree nor disagree or disagree or strongly disagree*	30 (8.2%)	5 (7.8%)
* Missing*	39 (10.7%)	9 (14.1%)
During my cancer treatment, I could find helpful information about being a LGBTQI + person with cancer		
* Strongly agree or Agree*	38 (10.4%)	5 (7.8%)
* Neither agree nor disagree or disagree or strongly disagree*	281 (76.8%)	48 (75.0%)
* Missing*	47 (12.8%)	11 (17.2%)
Has your provider talked to you about your post-treatment care plan (also referred to as a cancer survivorship plan), including things such as referrals to community services, reminders for future cancer screenings, and psychological support for adapting to life as a cancer survivor?		
* Yes*	203 (55.5%)	34 (53.1%)
* No*	103 (28.1%)	18 (28.1%)
* Missing*	60 (16.4%)	12 (18.8%)
If yes, does your post-treatment care plan include resources for LGBTQI + individuals?		
* Yes*	21 (10.3%)	1 (2.9%)
* No*	157 (77.3%)	29 (85.3%)
* Missing*	25 (12.3%)	4 (11.8%)
How important or unimportant is it to you that your post-treatment care plan includes information helpful to LGBTQI + individuals?		
* Very important or somewhat important*	193 (52.7%)	30 (46.9%)
* Neither important nor unimportant or somewhat unimportant or very important*	104 (28.4%)	17 (26.6%)
* Missing*	69 (18.8%)	17 (26.6%)
How welcoming or unwelcoming was the environment where you received cancer treatment?		
* Very welcoming or somewhat welcoming*	326 (89.1%)	55 (85.9%)
* Neither welcoming nor unwelcoming or somewhat unwelcoming or very unwelcoming*	26 (7.1%)	8 (12.5%)
* Missing*	14 (3.8%)	1 (1.6%)
How satisfied or dissatisfied were you with your overall cancer treatment experience?		
* Very satisfied or somewhat satisfied*	332 (90.7%)	53 (82.8%)
* Neither satisfied nor unsatisfied or somewhat satisfied or very unsatisfied*	17 (4.6%)	7 (10.9%)
* Missing*	14 (4.6%)	4 (6.3%)
Have you ever received cancer survivor social support?		
* Yes*	142 (38.8%)	31 (48.4%)
* No*	192 (52.5%)	22 (34.4%)
* Missing*	32 (8.8%)	11 (17.2%)
If yes, how welcoming or unwelcoming was the cancer survivor social support?		
* Very welcoming or somewhat welcoming*	122 (87.8%)	24 (82.8%)
* Neither welcoming nor unwelcoming or somewhat unwelcoming or very unwelcoming*	17 (4.6%)	5 (17.2%)
* Missing*	3 (2.1%)	2
How important or unimportant is it to you to be able to access LGBTQI + welcoming cancer survivor social support?		
* Very important or somewhat important*	233 (63.7%)	39 (60.9%)
* Neither important nor unimportant or somewhat unimportant or very unimportant*	99 (27.0%)	15 (23.4%)
* Missing*	34 (9.3%)	10 (15.6%)
How would you describe your current health?		
* Excellent/very good/good*	323 (63.4%)	36 (56.3%)
* Fair/poor*	84 (23.0%)	16 (25.0%)
* Missing*	50 (13.7%)	12 (18.8%)
I am able to access the resources I need to maintain or improve my health		
* Strongly agree or agree*	282 (77.0%)	46 (71.9%)
* Neither agree nor disagree or disagree or strongly disagree*	42 (11.5%)	9 (14.1%)
* Missing*	42 (11.5%)	9 (14.1%)

Although the majority of participants indicated that they could find helpful information about their cancer while undergoing cancer treatment (lesbian, 81.1%; bisexual, 78.1%), few participants indicated that they could find information about being an LGBTQ + person with cancer (lesbian, 10.4%; bisexual, 7.8%). Similarly, although half of the sample indicated that their provider had talked to them about their post-treatment care plan (lesbian, 55.5%; bisexual, 53.1%), over 75% indicated that their plan did not include resources for LGBTQI + individuals (lesbian, 77.3%; bisexual, 85.3%). Notably, they desired this information; many participants indicated that it was at least somewhat important that their post-treatment care plan includes information helpful to LGBTQI + individuals (lesbian, 52.7%; bisexual, 46.9%). Only 39% of lesbian breast cancer survivors and 48% of bisexual breast cancer survivors reported receiving cancer survivor social support. But they desired this support; more than 60% (lesbian, 63.7%; bisexual, 60.9%) indicated ability to access LGBTQI + welcoming cancer survivor social support was somewhat or very important.

### Associated survivorship experiences

Five key findings were consistent across lesbian and bisexual breast cancer survivors including differences in those who could not vs could access health-related resources at the time of being surveyed and differences in those who characterized their cancer treatment environment as less than welcoming vs welcoming.

1. Study participants who were unable to find helpful information about their cancer during the time of treatment were less likely to report being able to access resources needed to maintain or improve their health at the time of this survey than those who were able to find helpful information about their cancer (lesbian, 53% vs 90%, *p* < 0.001; bisexual, 40% vs 88%, *p* = 0.027).
2. Study participants who reported a less than welcoming cancer treatment environment were also less likely to report being able to access resources needed to maintain or improve their health at the time of this survey than those who experienced a welcoming cancer treatment environment (lesbian, 64% vs 88%, *p* = 0.002; bisexual, 40% vs 88%, *p* = 0.028).
3. Finally, study participants who reported a less than satisfactory cancer treatment experience were less likely to report being able to access resources needed to maintain or improve their health at the time of this survey than those who reported a satisfactory cancer treatment experience (lesbian, 41% vs 90%, *p* < 0.001; bisexual, 40% vs 88%, *p* < 0.001).
4. Unsurprisingly, those who characterized their cancer treatment environment as unwelcoming were less likely to be satisfied with their cancer treatment experience than those who characterized their treatment environment as welcoming (lesbian, 44% vs 95%, *p* < 0.001; bisexual, 43% vs 94%, *p* = 0.002).
5. Although more than half of respondents indicated their healthcare provider had discussed a post-treatment care plan with them, those who reported not having discussed a post-treatment care plan were more likely to be dissatisfied with their cancer treatment experience than those whose provider discussed a plan (lesbian, 11% vs 3%, *p* = 0.014; bisexual, 33% vs 0%, *p* = 0.002).

#### Findings unique to lesbian breast *cancer* survivors

There were three key findings that were specific to lesbian breast cancer survivors in our sample.

1. Those who reported being unable to access resources needed to maintain or improve their health at the time of this survey were more likely to prioritize accessing LGBTQI + welcoming cancer survivor social support than those with access (16% vs 6%, *p* = 0.027). Finally, those who reported being unable to access resources needed to maintain or improve their health at the time of this survey were more likely to be in poorer current health than those with access (26% vs 8%, *p* < 0.001).
2. Lesbian breast cancer survivors who reported not discussing a post-treatment care plan were more likely to report being unable to access resources needed to maintain or improve their current health at the time of this survey than those who reported having a provider that did talk to them about a post-treatment care plan (32% vs 4%, *p* < 0.001). These individuals were also more likely to report being in poorer current health than those who discussed a plan (46% vs 26%, *p* = 0.001). They were also more likely to report being unable to find helpful information about their cancer during treatment (63% vs 28%, *p* < 0.001) and report their cancer treatment environment as less than welcoming (68% vs 29%, *p* < 0.001) than those who reported discussing a post-treatment plan. Finally, those who reported not discussing a post-treatment plan were more likely to prioritize a post-treatment plan including information helpful to LGBTQI + (39% vs 22%, *p* = 0.002) than those who reported discussing a plan. Conversely, those who reported discussing a plan were more likely to have reported receiving cancer survivor social support than those who did not discuss a plan with their provider (51% vs 26%, *p* < 0.001).
3. Lesbian breast cancer survivors residing in the American South (US Census Region) were less likely to report a welcoming cancer treatment environment than those living in other regions of the USA (83%—South vs 98%—Northeast, 97%—Midwest, 89%—West, *p* = 0.007). They were also less likely to report having received helpful information about their cancer than those living in other regions of the USA (83%—South vs 93%—Northeast, 97%—Midwest, 93%—West, *p* = 0.046).

### Findings unique to bisexual breast *cancer* survivors

There were two significant findings unique to the sample who identified as bisexual breast cancer survivors. The first was that those who indicated they could not find helpful information about their cancer during treatment were more likely to be dissatisfied with their overall cancer treatment experience than those who reported that they could find helpful information (80% vs 4%, *p* < 0.001). The second significant finding was that those who perceived their cancer treatment environment as less than welcoming were more likely to have pursued cancer survivor social support (71% vs 51%, *p* = 0.036) than those who perceived their cancer treatment environment as welcoming.

### Qualitative findings

Eighty-nine lesbian and 16 bisexual breast cancer survivors provided responses to the open-ended question: *Is there anything else you would like to share with us about developing post-treatment care plans for LGBTQI* + *individuals*? Several qualitative themes were common in their reaction. These included the following: (1) Holistic population-tailored post-treatment cancer survivorship resources were important but lacking in many cases; (2) connection with supportive others is a critical cancer survivorship need; (3) acknowledgement and affirmation of sexual orientation are important.

#### Holistic population-tailored post-treatment *cancer* survivorship resources were important but lacking in many cases

Post-treatment resources are primarily geared toward monitoring for disease recurrence and are created with a cis-gender and heteronormative lens. Post-treatment care plans need to address holistic topics, and resources that do not alienate survivors but rather promote inclusiveness are needed. Representative quotes included the included:

It is my dream that hospitals would refer patients and have an inclusive physical space to treat the whole person with art, music, writing therapy in a complementary way or online resources. I do feel that more resources need to be provided specifically for the LGBTQI community. – Lesbian Breast Cancer Survivor.

### Don’t know about post treatment plans. Didn’t have one except yearly checkups. – Bisexual Breast *Cancer* Survivor

I think post-treatment care plan should ensure that the patient has access to safe housing, adequate nutrition, and appropriate follow up medical care. Community services needs should be included in the discharge from care instructions to address any issues are identified. – Lesbian Breast Cancer Survivor.

### The post-treatment plan is not wholistic. It is medical only…screenings and follow up appts – Lesbian Breast *Cancer* Survivor

It would just be nice to know if/that they exist and how to connect. – Lesbian Breast Cancer Survivor

There is very little out there that is easily found or accessed. – Lesbian Breast Cancer Survivor

I would have loved something like that.—Bisexual Breast Cancer Survivor

Make resources more available. Lesbian Breast Cancer Survivor

It’s pretty much non-existent. – Lesbian Breast Cancer Survivor.

I find that in general cancer survivorship is woefully under-funded and there are not enough programs to offset the life-long side effects that come by being one of the "lucky ones." – Lesbian Breast Cancer Survivor.

I was given no plan and I’ve been struggling since finishing treatment. Luckily, I do therapy twice a week. I feel worse after cancer than during—and there is no support that I know of – Lesbian Breast Cancer Survivor.

I would like to see it mentioned – Bisexual Breast *Cancer* Survivor

Resources are hard to find. – Lesbian Breast *Cancer* Survivor

Health and body care (nutrition, exercise) information and support [are needed]. – Lesbian Breast *Cancer* Survivor

He [my doctor] screens me every year for signs of *cancer*, but no talk of support groups or psychological assistance, which would have been appreciated. – Lesbian Breast *Cancer* Survivor

#### Connection with supportive others is a critical *cancer* survivorship need

Participants repeatedly mentioned the importance of connecting and being in community with those who could provide encouragement. Representative quotes included the following:

I just need support. Having support from my own peers who are gay would be great, but support in general is big. When it hasn’t been there, it’s been very hard. Very very hard. – Lesbian Breast Cancer Survivor.

We need a more open-minded network and MORE support groups! – Bisexual Breast *Cancer* Survivor

I wish I could find a group again. – Lesbian Breast *Cancer* Survivor

I have Lesbian friends who are *cancer* survivors, so they have been a great support to me. -Lesbian Breast *Cancer* Survivor

I need like minds. If you don’t have them, seek them out. – Lesbian Breast *Cancer* Survivor

It was very helpful to me to connect with other lesbian breast *cancer* survivors—used to do this regularly at Womyn’s festivals – Lesbian Breast *Cancer* Survivor

I think I’d like to connect with other lesbian breast cancer survivors. I think they would understand better than my straight friends why it doesn’t bother me not to have breasts. Lesbian Breast Cancer Survivor.

#### Acknowledgement and affirmation of sexual orientation are important

Participants discussed the significance of feeling seen by their healthcare providers and healthcare facilities. Representative quotes included the following:

I don’t know what to say. I guess that what I experienced was ‘supportive’ care and it was fine. ‘Affirming’ care would / could be amazing. – Lesbian Breast Cancer Survivor.

That it be acknowledged. They think the gold standard is to treat everyone the same… by heterosexual standards. – Bisexual Breast *Cancer* Survivor

It’s important to take away the ‘friction’ of wondering if recommended groups, medical practices, and services are LGBT-friendly or not. If I’m feeling vulnerable because of cancer, I won’t have the psychological energy to, say, join a cancer support group not knowing whether they are going to support me fully or not. – Lesbian Breast Cancer Survivor.

Include conversations about sex, and please tailor it to my identity as a femme. – Bisexual Breast *Cancer* Survivor

In my experience it is pretty lonely. All my medical providers are straight. The rare times I have talked to a survivor she has been straight too. – Lesbian Breast *Cancer* Survivor

First, the care provider has to actually know if their patient is LGBTQIA + so they need to do that first (if the patient is comfortable disclosing). – Bisexual Breast *Cancer* Survivor

## Discussion

### Summary

Our findings indicate that although many LB breast cancer survivors report their cancer survivorship experiences as positive, enthusiasm for this finding is tempered as “not horrible” experiences may be conflated as “positive experiences.” Affirming, rather than simply accepting, care is needed in order to help reduce disparities [22]. Importantly, over 75% of our sample indicated they could not find helpful information about being a LGBTQI + person with cancer during their treatment. This is consistent with other studies highlighting a gap in resources and support groups with those that do exist being largely heteronormative [23, 24]. Material and programs that do not assume a solely heterosexual audience would be welcomed. Although the “treatment” may not necessarily need to be different, support resources that are actually, rather than nominally, helpful are important. Over 75% of our sample indicated that their cancer survivorship plan did not include resources for LGBTQI + individuals, consistent with previous studies [22, 24]. Experiencing a welcoming cancer treatment environment is of utmost importance and is associated with cancer care satisfaction. Another study demonstrated that perceived discrimination and social support were as important predictors for lesbian and bisexual breast cancer survivors’ quality of life [11]. Also associated with cancer care satisfaction is receiving a clear and comprehensive survivorship care plan. Dunne and colleagues’ [25] study exploring the 4R Oncology Model supports the utilization of tailored resources to encourage communication during and after cancer treatment. Our study demonstrated that having a satisfactory cancer treatment experiencing and receiving information about one’s cancer is associated with being able to access relevant health resources post-treatment. Essentially, those who have a negative experience during treatment are more likely to continue having a negative experience after treatment. This may have far-reaching consequences not only for LB breast cancer survivors’ quality of life but also for the willingness of survivors to engage in critical cancer recurrence screening. As existing data is limited, further study is needed to better understand this aspect of care in this population.

### Limitations

While having many strengths, our study has several limitations. First, the parent survey targeted all SGM cancer survivors so the amount of detailed information gained may be limited since it was not tailored to breast cancer survivors only, and this also limits the ability to explore individual differences within the breast cancer survivor population. Another limitation is that the open text information gathered was limited to one question rather than a comprehensive interview or focus group about survivorship experiences, thus limiting the information provided. Additionally, the open-ended question was asked toward the end of the survey, so survey fatigue may have further limited the information provided as well as contributed to missing responses. An important limitation to highlight is that because of the survey’s cross-sectional design, only association and not causality can be determined.

### Future study and next steps

Future studies should focus on development and testing of population-specific resources to include within survivorship care plans. These resources should aim to help patients feel “less alone.” In their post-treatment experiences, studies on best practices for helping patients feel “seen” over the continuum of cancer care, rather than only at one isolated point, are needed. While visible symbols signaling a welcoming environment (e.g., rainbows, pride flags) are important, research should focus on additional strategies (e.g., care navigation) to reduce disparities in this population. Available national resources for this community of survivors include the LGBT Cancer Network and the American Cancer Society.

## Supplementary Information

Below is the link to the electronic supplementary material.

Supplementary file1 (DOCX 15.9 KB)

## Data Availability

Data are available upon reasonable request and IRB approval.
