# Supplementary material for: Lesbian and bisexual breast cancer survivors’ post-treatment resource needs
Source: J Cancer Surviv. 2024 Aug 5;20(1):236–44. doi: 10.1007/s11764-024-01650-y (PMC12906544; doi:10.1007/s11764-024-01650-y)
Supplement: Supplementary file 1 — Supplementary file1 (DOCX 15.9 KB) [file 11764_2024_1650_MOESM1_ESM.docx]

**Supplemental Table. Survey Questions related to Cancer Survivorship**

- During my cancer treatment, I could find helpful information about my cancer.

Strongly agree | Agree | Neither agree nor disagree | Disagree | Strongly disagree

Prefer not to answer

- During my cancer treatment, I could find helpful information about being a LGBTQI+ person with cancer.

Strongly agree | Agree | Neither agree nor disagree | Disagree | Strongly disagree

Prefer not to answer

- Has your provider talked to you about your post-treatment care plan (also referred to as a cancer survivorship plan), including things such as referrals to community services, reminders for future cancer screenings, and psychological support for adapting to life as a cancer survivor?

Yes | No | Prefer not to answer

- Does your post-treatment care plan include resources for LGBTQI+ individuals?

Yes | No | Prefer not to answer

- How important or unimportant is it to you that your post-treatment care plan includes information helpful to LGBTQI+ individuals?

Very important | Somewhat important | Neither important nor unimportant | Somewhat unimportant | Very unimportant

Don’t know/Prefer not to answer

- How welcoming or unwelcoming was the environment where you received cancer treatment?

Very welcoming | Somewhat welcoming | Neither welcoming nor unwelcoming | Somewhat unwelcoming | Very unwelcoming;

Don’t know/Prefer not to answer

- How satisfied or dissatisfied were you with your overall cancer treatment experience?

Very satisfied | Somewhat satisfied | Neither satisfied nor unsatisfied | Somewhat unsatisfied | Very unsatisfied

Don’t know/Prefer not to answer

- Have you ever received cancer survivor social support?

Yes | No | Prefer not to answer

- If yes, how welcoming or unwelcoming was this cancer survivor social support?

Very welcoming | Somewhat welcoming | Neither welcoming nor unwelcoming | Somewhat unwelcoming | Very unwelcoming

Don’t know/Prefer not to answer

- How important or unimportant is it to you to be able to access LGBTQI+ welcoming cancer survivor social support?

Very important | Somewhat important | Neither important nor unimportant | Somewhat unimportant | Very unimportant

Don’t know/Prefer not to answer

- How would you describe your current health?

Excellent | Very Good | Good | Fair | Poor

Don’t know/Prefer not to answer

- I am able to access the resources I need to maintain or improve my health.

Strongly agree | Agree | Neither agree nor disagree | Disagree | Strongly disagree

Prefer not to answer

- Is there anything else you would like to share with us about developing post-treatment care plans for LGBTQI+ individuals (open text)
